# Ecological Assessment of Clinicians’ Antipsychotic Prescription Habits in Psychiatric Inpatients: A Novel Web- and Mobile Phone–Based Prototype for a Dynamic Clinical Decision Support System

**DOI:** 10.2196/jmir.5954

**Published:** 2017-01-26

**Authors:** Sofian Berrouiguet, Maria Luisa Barrigón, Sara A Brandt, George C Nitzburg, Santiago Ovejero, Raquel Alvarez-Garcia, Juan Carballo, Michel Walter, Romain Billot, Philippe Lenca, David Delgado-Gomez, Juliette Ropars, Ivan de la Calle Gonzalez, Philippe Courtet, Enrique Baca-García

**Affiliations:** ^1^ Department of Psychiatry Brest Medical University Hospital at Brest Brest France; ^2^ UMR CNRS 6285 Lab-STICC Institut Mines-Telecom Brest France; ^3^ ERCR SPURBO Université de Bretagne occidentale Brest France; ^4^ Department of Psychiatry Fundacion Jimenez Diaz Hospital Madrid Spain; ^5^ Department of Psychiatry, Icahn School of Medicine at Mount Sinai New York, NY United States; ^6^ IMT Atlantique UMR CNRS 6285 Lab-STICC Technopôle Brest Iroise Brest France; ^7^ Departamento de Estadistica, Universidad Carlos III de Madrid Madrid Spain; ^8^ LaTIM - INSERM UMR 1101 Brest Medical University Hospital Brest France; ^9^ Cabaro Soluciones SA Valladolid Spain; ^10^ INSERM U1061 Université de Montpellier Montpellier France

**Keywords:** clinical decision-making, antipsychotic agents, software, mobile applications, off-label use, prescriptions

## Abstract

**Background:**

Electronic prescribing devices with clinical decision support systems (CDSSs) hold the potential to significantly improve pharmacological treatment management.

**Objective:**

The aim of our study was to develop a novel Web- and mobile phone–based application to provide a dynamic CDSS by monitoring and analyzing practitioners’ antipsychotic prescription habits and simultaneously linking these data to inpatients’ symptom changes.

**Methods:**

We recruited 353 psychiatric inpatients whose symptom levels and prescribed medications were inputted into the MEmind application. We standardized all medications in the MEmind database using the Anatomical Therapeutic Chemical (ATC) classification system and the defined daily dose (DDD). For each patient, MEmind calculated an average for the daily dose prescribed for antipsychotics (using the N05A ATC code), prescribed daily dose (PDD), and the PDD to DDD ratio.

**Results:**

MEmind results found that antipsychotics were used by 61.5% (217/353) of inpatients, with the largest proportion being patients with schizophrenia spectrum disorders (33.4%, 118/353). Of the 217 patients, 137 (63.2%, 137/217) were administered pharmacological monotherapy and 80 (36.8%, 80/217) were administered polytherapy. Antipsychotics were used mostly in schizophrenia spectrum and related psychotic disorders, but they were also prescribed in other nonpsychotic diagnoses. Notably, we observed polypharmacy going against current antipsychotics guidelines.

**Conclusions:**

MEmind data indicated that antipsychotic polypharmacy and off-label use in inpatient units is commonly practiced. MEmind holds the potential to create a dynamic CDSS that provides real-time tracking of prescription practices and symptom change. Such feedback can help practitioners determine a maximally therapeutic drug treatment while avoiding unproductive overprescription and off-label use.

## Introduction

### From Electronic Health Records to mHealth Applications

Over the last decade, management of patients in hospitalization units has been supported by the emergence of electronic health records (EHRs) [1]. This software facilitates portability and processing of pertinent health and pharmacological treatment information [2]. These systems can support prescription practice and help practitioners determine maximally therapeutic pharmacological treatments while avoiding pitfalls such as off-label use, polypharmacy, and overly high dosages. Moreover, the emergence of electronic prescribing or e-prescribing devices with clinical decision support systems (CDSSs) has significantly reduced diagnosis and prescription error rates [3]. However, it remains difficult to extract clinically relevant information from current CDSS tools, as these data are often not tailored to the exact needs of the patient and clinician [4]. The availability of mobile phones and other handheld computers provides the opportunity to improve prescription practices in institutions where e-prescribing systems are not yet available. For example, Web-based and mobile phone–based programs permit the gathering of naturalistic data that can be processed immediately and provide instantaneous decision-making assistance to clinicians [5,6]. Overall, the appearance of these devices in medical practice has heralded the mobile health (mHealth) era, which in turn falls under the umbrella of electronic health (eHealth), where mobile devices are used to advance public health [7]. The combination of high levels of mental illness and high levels of mobile phone usage worldwide highlights the potential for mH^2^ interventions (ie, mHealth mental health interventions) [8].

Wirelessly connected technologies have also increased communication and data transfer between clinicians and their patients, which further helps achieve these mHealth goals [9]. The processing of naturalistic data is especially critical given the enormous complexity of individual conditions [10]. Data mining techniques can also allow for automatic extraction of meaningful data from large clinical databases, which can help answer important treatment and outcome questions and refine best practices. Moreover, this data mining can help develop algorithms and guidelines to help care providers who require assistance [11]. These algorithms may be of particular interest to prescribers and can be used to improve the incorporation of prescription guidelines into clinical practice [12].

### Challenges in Managing Psychopharmacological Treatments

However, despite these technological advances, the management of psychopharmacological treatment still grapples with many challenges. Antipsychotics are widely prescribed in psychiatric inpatient units and, as a result, off-label use is common. Although they are mostly prescribed in schizophrenia spectrum and related disorders, antipsychotics are also used off-label in a range of chronic diseases and they have been utilized as augmentation for depressive disorder [13], autism spectrum disorders [14], or off-label use, which is controversial but not uncommon [15]. For example, one study conducted across 7 provinces within Spain showed that antipsychotics were not only used in schizophrenia (22.8%) but also in other psychiatric disorders such as bipolar disorder (14.4%), depressive disorders (12.5%), personality disorders (9%), substance use disorders (1.3%), and dementia (4.5%), with 32.8% considered off-label uses [16]. Antipsychotic polypharmacy (APP) is also controversial yet quite common. APP is the use of 2 or more antipsychotics concurrently by a single patient. APP is commonly used against general clinical guideline recommendations [17]. A recent systematic review of APP prevalence between 1970 and 2009 found in a sample of 1,418,163 patients with mental disorder (82.9% with a diagnosis of schizophrenia) a median APP prevalence of 19.6% across different geographical regions, ranging from 6% to 90%, with higher median prevalence in Asia (32%) and Europe (23%) compared with North America (16%) and Oceania (16.4%) [18]. APP differs according to treatment setting, requiring more extended use in greater illness severity, such as that in inpatient settings [19,20]. In Spanish inpatient settings, APP is common with 47.1% of patients in a psychiatric hospital [21] and figures between 40% and 50% in psychiatric brief hospitalization units [22]. These studies highlight the discrepancy between guidelines and the real-world treatment. We can observe a paradigm shift in APP from discouraging all uses of polypharmacy to determining patient profiles that could benefit from polypharmacy [23]. In a naturalistic observational study, Gaviria et al [24] analyzed existing EHRs to collect data on prescription habits in a sample of 1765 patients with schizophrenia. Out of the sample of 1765 patients, 505 (28.6%) were receiving treatment with antipsychotic monotherapy, whereas 1229 (69.6%) were receiving 2 or more antipsychotics. Another concern regarding antipsychotic prescription is the use of antipsychotics above their recommended doses. Overly high dosage can increase risk for adverse reactions and increases treatment cost without clear evidence of added therapeutic benefit [24]. This practice, although prevalent in clinical settings, is discouraged in clinical guidelines [25]. These challenges highlight the importance of exploring innovative prescription monitoring methods for the field of mental health.

### Toward a Mobile Clinical Decision Support System

Given the many proven benefits of EHRs for clinical practice, EHRs may also provide a possible solution to the issues that exist with antipsychotic prescription habits. Specifically, EHRs are a major source of structured data that can provide useful and ecologically valid insights into how antipsychotics are prescribed. These EHR systems, however, presented many limitations that are related to the methodology of such studies. First, conducting clinical research using data produced by EHRs can be challenging, as the timing, quality, and comprehensiveness of the clinical data often do not meet the rigorous standards of clinical research. Furthermore, owing to the architecture of traditional EHRs, data cannot be processed instantaneously to deliver a CDSS. Before delivering a CDSS, data must be deidentified as well as statistically analyzed, which has not yet been automated by software into an instantaneous process. Notably, most studies that use EHRs to describe antipsychotic habits implement retrospective methods [26]. As a result, researchers and clinicians miss the opportunity to process gathered data in the moment and use these data for clinical decision making. Finally, EHRs are usually only accessible as expensive commercial software packages, which precludes assessment of inpatients treated by institutions outside of large, mainstream health care institutions.

Web-based and mobile phone–based prescription management tools exhibit numerous advantages over these expensive, mainstream EHR software packages. Specifically, the low development cost and increasing popularity of mHealth apps (ie, health-related software applications) have made them particularly accessible across both large and small psychiatric care settings. In addition, mHealth apps often feature simple interfaces, can be used wirelessly from any location with a cell signal, and provide adequate computing power to provide CDSS capability. For the most part, these mHealth apps in mental health have been directly marketed to either consumers or small clinical practice settings. A review of mobile phone apps for schizophrenia found only 5 studies of mobile phone apps for patients with schizophrenia. All examined feasibility, and one assessed the preliminary efficacy [27]. For example, Nicholas et al [7] showed that the contents of currently available apps for bipolar disorder are not in line with practice guidelines or established self-management principles.

Taking into consideration the strengths and pitfalls of each of these strategies, our aim was to develop a Web application that could monitor prescription habits in psychiatric inpatient units. This study presents the preliminary step in the development of a CDSS. Our hypothesis was that a Web application developed for this study may be able to describe clinicians’ prescriptions in a sample of inpatients. The objective of this study was to describe via the MEmind Web application the prescription habits of antipsychotics in a naturalistic inpatient setting focusing on off-label uses and APP.

The ultimate goal of this MEmind prototype is to allow physicians to provide more effective care while better adhering to clinical guidelines.

## Methods

### Study Design

This pilot study was a 5-month, multicenter, nonrandomized, and observational feasibility study. Participants were adults admitted in 2 brief psychiatric inpatient units (Fundación Jiménez Díaz Moncloa Hospital and Pontones Hospital, Madrid, Spain).

### Setting

The 2 brief psychiatric inpatient units are part of the Department of Psychiatry of the Fundación Jiménez Díaz Hospital, which belongs to the National Health Services and provides medical coverage financed by taxes to a catchment area of ~800,000 people. A total of 4 psychiatrists are in charge of the 20 beds in each of these units. Roughly 14 nurses cover 3 work shifts in both units. Patients admitted were treated as usual. The psychiatrist in charge of the patient proposed participation in the study after verification of the inclusion criteria.

### Inclusion and Exclusion Criteria

Inclusion criteria were either males or females, aged 18 years or older, who were admitted to a psychiatric inpatient unit, and who gave written informed consent. Participants were excluded from the study if they were younger than 18 years, incarcerated, under guardianship, were enrolled in other trials, or were in emergency situations where their state of health did not allow for obtaining written informed consent.

### Study Procedure

**Figure 1:**
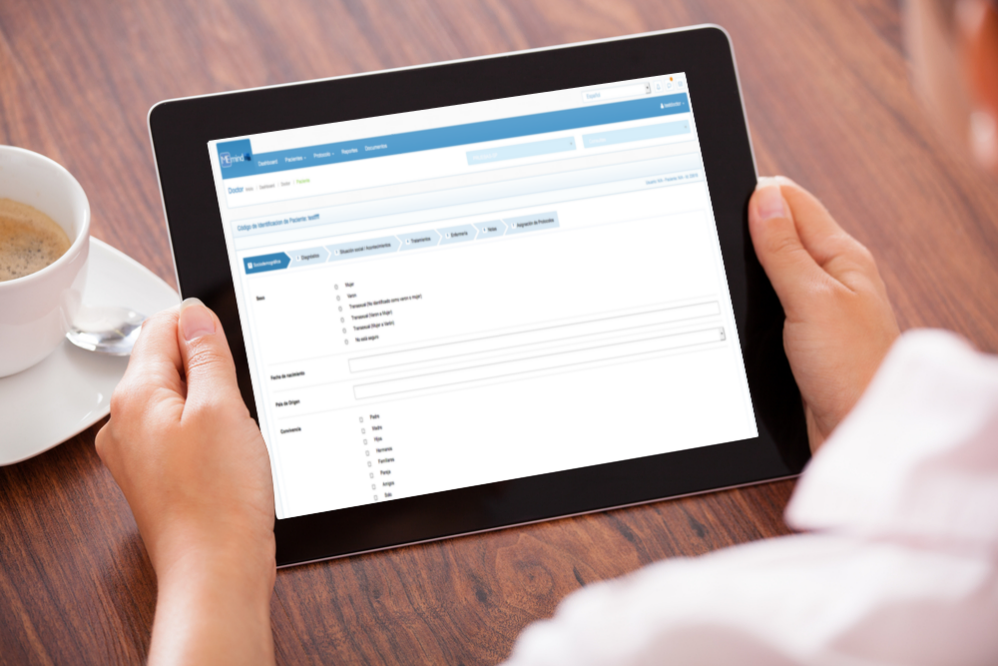
View of the MEmind Web application.

During their hospitalization in the psychiatric unit (PU), all patients were assessed with the MEmind Web application after giving written informed consent. The MEmind application was developed for the study by an industrial partner and is available online for download via a secure link provided to the participant via email. The application was designed to gather observational data and to perform ecological momentary assessment (EMA). It has 2 distinct views: the clinician view and the patient view. The “clinician” view is designed to be used by doctors and nurses during their clinical rounds and during their medical or nursing visits (see Figure 1, which displays the MEmind Web application). MEmind was also designed to capture all the data typically gathered during a standard medical evaluation, including sociodemographic, diagnostic, and pharmacological treatment information. MEmind’s design is based on commonly stored information in mental health management. To this end, the care providers can customize the software to add a number of large relevant scales to suit their individual needs (eg, the specific needs of research vs clinical settings). At the time of discharge, we collected patients’ sex, age, diagnosis, and treatment types during their stay in the psychiatric unit. As the study was performed in Spanish mental health centers, the Spanish version of the tutorials for mental health professionals was used. In addition to the clinician view, the MEmind program features a patient view that allows patients to track their symptoms using EMA techniques. This feature was not assessed in our study. Medication compliance was not assessed in this study. For the purposes of this study, we monitored patients’ diagnoses and clinicians’ prescriptions. The only users who accessed the app were the clinicians who recorded their prescriptions directly into MEmind’s clinician interface. Clinicians could access the Web application either from a computer or their personal mobile phone.

Clinical diagnoses and treatment prescription were conducted during hospital admission as part to routine psychiatric evaluations that incorporated data from medical records, other research assessments, and clinical interviews. All diagnoses recorded into the MEmind app were coded according to the *International Classification of Diseases, Tenth Revision*, for mental disorders alongside the data from these aforementioned psychiatric evaluations.

### Outcome Measures

Antipsychotic medication treatments were recorded into the MEmind app and then classified according to the Anatomical Therapeutic Chemical (ATC) classification system and the defined daily dose (DDD). For each patient, the treatment management function of the Web application calculated the average of daily dose prescribed for antipsychotics (N05A ATC code), prescribed daily dose (PDD), PDD 95% CI, and the mean PDD to DDD ratio. Although ATC classification includes lithium and antipsychotics under the N05A code, we chose to include only antipsychotics for the purposes of this study. To compare dosages of various antipsychotics, we used a fixed unit of measurement calculated by dividing the PDD by the DDD. A PDD/DDD ratio greater than 1.5 was defined as excessive dosing [28]. All analyses were conducted using SPSS version 22.0 (IBM Corporation).

### Ethical Considerations

The research was in compliance with the Code of Ethics of the World Medical Association (Declaration of Helsinki) and the standards established by the institutional review board and granting agency. All the participants provided written informed consent after the complete description of the study. Previously, the research protocol was approved by the Ethics Committee of Fundación Jiménez Díaz, Madrid.

## Results

### Sample Characteristics

A total of 359 patients received brief psychiatric care in the 2 psychiatric inpatient units from June 2014 to October 2014. Among them, 353 patients were evaluated with the MEmind application. Of the 6 patients who did not participate in the study, 1 patient refused to participate and 5 patients did not receive the proposal to participate. The distribution of the main psychiatric disorders according to age and sex is presented in Table 1.

Out of our total sample, 243 patients had 1 psychiatric diagnosis, with schizophrenia spectrum and related disorders being the most frequent diagnoses. Other diagnoses were comorbid diagnoses and were considered secondary to their psychotic disorder diagnoses. Antipsychotics, alone or in combination with other psychotropic drugs, were used in 217 of the 353 patients (61.5%). Of the 217 patients, 137 (63.2%) were administered pharmacological monotherapy and 80 (36.8%) were administered polytherapy (for details, see Table 2).

**Table 1:** Age and sex distribution of psychiatric disorders.

Psychiatric disorder	Patients with diagnosis, n (%)	Age, %					Sex, %		
		18-35 years	35-50 years	50-65 years	>65 years	*P* value^a^	Female	Male	*P* value^a^
Substance use disorders	79 (22.4)	19.3	47.4	33.3	0	.02	51.8	48.2	.41
Schizophrenia and other psychoses	134 (38)	28.4	37.9	24.2	9.5	.83	42.5	57.5	.79
Mood disorders	100 (28.3)	25.7	23.0	35.1	16.2	.009	54.0	46.0	.238
Personality disorders	70 (19.8)	35.4	47.9	16.3	0	.007	52.9	47.1	.505
Total (N=353)		27.3	35.5	27.3	9.8		48.7	51.3	

^a^The *P* value was calculated using the chi-square test.

**Table 2:** Antipsychotic medication use according to diagnosis.

Diagnosis		Patients with diagnosis, n (%)	Antipsychotic use	Antipsychotic monotherapy	Antipsychotic polytherapy
**One diagnosis (n=243)**					
	Substance use disorders	16 (4.5)	8	6	2
	Schizophrenia and other psychosis	101 (28.6)	101	62	39
	Mood disorders	69 (19.5)	51	33	18
	Anxiety-related disorders	24 (6.8)	5	4	1
	Personality disorders	21 (5.9)	12	10	2
	Rest of the diagnoses	12 (3.4)	0	0	0
**Comorbidity (n=110)**					
	Organic disorders + mood disorders	5 (1.4)	2	1	1
	Substance use disorders + schizophrenia and other psychosis	10 (2.8)	9	4	5
	Substance use disorders + mood disorders	6 (1.7)	6	4	2
	Substance use disorders + personality disorders	7 (2.0)	9	4	5
	Schizophrenia and other psychosis + personality disorders	7 (2.0)	6	3	3
	Mood disorders + personality disorders	6 (1.70)	4	2	2
	Anxiety-related disorders + personality disorders	9 (2.5)	4	4	0
	Other combinations	60 (17)	0	0	0
Total		353	217	137	80

### Antipsychotic Medication Use Pattern

The frequencies of prescription according to diagnoses, as a unique or comorbid condition, were as follows: Of the 353 patients, 118 (33.4%, 118/353) patients had a diagnosis of schizophrenia and other psychosis (F20-F29), 86 (24.3%, 86/353) had a diagnosis of mood disorders (F30-F39), 50 (14.1%, 50/353) had a diagnosis of personality disorders (F60-F69), and 33 (9.3%, 33/353) had a diagnosis of anxiety-related disorders (F40-F49; see Table 2).

Antipsychotics were prescribed to 217 patients corresponding to a total of 365 antipsychotics prescriptions. In 62 (29.2%) patients, antipsychotics were the only psychotropic drug prescribed; in 40 (18.9%) patients 1 antipsychotic was prescribed and in 22 (10.4%) patients 2 antipsychotics were prescribed. For the remaining 155 patients, antipsychotics were used in combination with other psychotropic drugs. Thus, only 18.9% of our sample were patients in a pure monotherapy antipsychotic regimen. Table 3 presents the different antipsychotics prescribed, ATC/DDD classification, and doses used in our clinical practice. The antipsychotics used in excessive doses were amisulpride, olanzapine, risperidone, and paliperidone (both their oral and long-acting injectable forms). On the other hand, levomepromazine was used in the lowest dose followed by clotiapine and quetiapine.

The antipsychotics used more frequently in APP were clozapine (81.8%), clotiapine (81.8%), and amisulpride (70.6%) through oral administration and fluphenazine (100%), zuclopenthixol acufase (100%), and zuclopenthixol depot (83.3%) through long-acting injectable forms (for details, see Multimedia Appendices 1 and 2).

**Table 3:** Anatomical Therapeutic Chemical classification with defined daily dose, prescribed daily dose values, and prescribed daily dose to defined daily dose ratio of antipsychotics prescribed.

No. of prescriptions (N=365)	Drug	ATC^a^ code	DDD^b^ (mg)	Median PDD^c^ (mg)	Mean PDD (mg)	PDD (mg) 95% CI	Mean PDD/DDD^d^
17	Amisulpride^e^	N05AL05	400	800	811.76	652.4-971.1	2.03
37	Aripiprazole^e^	N05AX12	15	15	20.54	16.7-24.4	1.37
25	Asenapine^e^	N05AH05	20	10	14.4	11.3-17.5	0.72
11	Clotiapine^e^	N05AH06	80	40	35.45	24.4-46.5	0.44
11	Clozapine^e^	N05AH02	300	350	345.45	284.2-406.7	1.15
3	Fluphenazine^f^	N05AB02	1	0.89	1.19	0.61-1.78	1.19
7	Haloperidol^f^	N05AD01	8	5	7.63	2.4-12.9	0.95
3	Levomepromazine^f^	N05AA01	300	50	58.33	42.0-74.7	0.19
36	Olanzapine^e^	N05AH03	10	10	15.76	12.5-19.1	1.58
22	Paliperidone^f^	N05AX13	6	10.5	11.59	9.5-13.7	1.93
63	Long-acting paliperidone^f^	N05AX13	2.5	3.57	4.79	4.34-5.24	1.92
38	Quetiapine^f^	N05AH04	400	150	228.29	153.5-303.1	0.57
66	Risperidone^f^	N05AX08	5	6	7.61	6.5-8.7	1.52
1	Long-acting risperidone^e^	N05AX08	2.7	7.14	7.14	7.14-7.14	2.64
10	Tiapride^e,f^	N05AL03	400	300	290	244.3-335.7	0.73
1	Ziprasidone^f^	N05AE04	80	120	120	120.0-120.0	1.50
2	Zuclopenthixol acufase^f^	N05AF05	30	25	25	25-25	0.83
12	Zuclopenthixol depot^f^	N05AF05	15	9.5	10.3	8.8-11.9	0.69

^a^ATC: Anatomical Therapeutic Chemical.

^b^DDD: defined daily dose.

^c^PDD: prescribed daily dose.

^d^Mean PDD to DDD ratio.

^e^Oral administration.

^f^Injectable administration.

## Discussion

### Principal Findings

This study described a reproducible method for performing naturalistic prospective prescription analysis via a Web- and mobile phone–based application prototype, MEmind. This observational study was the first step in the development of a CDSS that may help care providers better monitor their prescriptions and make decisions regarding pharmacological treatment. In this study, we were able to identify polypharmacy, overly high dosage, and off-label use in a psychiatric inpatient setting. We found that APP was used in 35.8% of the patients in our 2 brief psychiatric inpatient units, with clozapine as the oral drug most used in APP and fluphenazine as the long-acting injection drug most used in APP. Antipsychotics were used mostly in schizophrenia spectrum and related psychotic disorders, but they were also prescribed in other nonpsychotic diagnoses. Risperidone and paliperidone, in both their oral and long-acting presentation, were the most prescribed antipsychotics. With respect to dosing, with the exception of only one prescription of long-acting risperidone, amisulpride was the antipsychotic prescribed at highest doses, whereas levomepromazine was the antipsychotic prescribed at lowest doses.

In our sample, the oral antipsychotics most used in APP regimen were clozapine, clotiapine, and amisulpride. One likely explanation for these findings regarding clotiapine in APP is that clotiapine is not principally used for its antipsychotic properties but rather for its hypnotic properties; this interpretation is supported by clotiapine’s PDD/DDD ratio of 0.44. Clozapine was used in APP in 81.8% of cases. Out of 11 patients who were administered clozapine, 9 patients received pharmacological polytherapy and only 2 patients received monotherapy with a PDD/DDD ratio of 1.15. Amisulpride was used in APP in 70.6% (12/17) of cases. Our use of these antipsychotics is consistent with their pervasive clinical use in our country [21] and worldwide [29].

Clinical guidelines recommend the use of clozapine in APP only for ultraresistant patients with schizophrenia [22]. This will reduce clozapine dose, minimize adverse effects and allow for the use of amisulpride APP to be the rule rather than the exception [29]. Moreover, a very common antipsychotic combination is the clozapine augmentation with amisulpride in patients with ultraresistant schizophrenia.

Long-acting antipsychotics were rarely used with first-generation antipsychotics but were used most frequently in APP. Second-generation antipsychotics, however, were used in pharmacological monotherapy and polytherapy in the same proportion. In our sample, long-acting paliperidone has replaced long-acting risperidone (used in only 1 patient) and is used in APP in almost 50% of cases. The use of paliperidone in this inpatient setting probably represents a fluctuation period during the hospitalization.

When observing the range of doses used in our sample, we noticed that the drugs used in higher doses were amisulpride (PDD/DDD = 2.03) as well as paliperidone in its long-acting presentation (PDD/DDD = 1.92) and oral presentation (PDD/DDD = 1.93). Both of these drugs are antipsychotics with a high affinity for dopamine D_2_ receptor blockade. As a D_2_ receptor binding of 70% is necessary for therapeutic benefits [30], high doses of these antipsychotics are commonly used to better reach this level of binding. Given that many patients arrive at inpatient settings with severe psychopathology, clinicians may attempt to use pharmacotherapies at higher dosages to stabilize patients in shorter amounts of time. On the other hand, levomepromazine, clotiapine, and quetiapine were the antipsychotics used at the lowest doses. Specifically, their PDD/DDD ratios were less than 0.5, which likely reflects levomepromazine and clotiapine being prescribed for nonpsychotic symptoms (such as insomnia or anxiety) and low doses of quetiapine being prescribed for bipolar depression [30].

### Limitations

To improve acceptance among care providers, diagnoses were provided by psychiatrists rather than being obtained as the result of a structured clinical interview, such as the Structured Clinical Interview for DSM-5 (SCID-5). As shown in other studies, the implementation of EHRs or CDSSs may increase clinician workload [26]. In order to ease the burden and improve acceptance of the study procedure among clinicians, we did not include in the baseline assessment a structured interview of participants. This “as usual” approach of conducting is also consistent with the noninterventional setting of our study. However, to further assess the effect of implementation of the CDSS on patients’ clinical outcomes, a standard assessment will be performed in a forthcoming study.

In this study, we did not report the effect of MEmind on prescription habits. Changes in prescription behaviors have been reported by other studies describing the implementation of an e-prescribing tool [31]. It could have been of interest to report the effect of having instantaneous feedback from MEmind concerning off-label use. This, however, would have required a distinct methodology relying on a randomized controlled trial and a larger sample, which does not fit with a feasibility study.

### Perspectives

This Web- and mobile phone–based application allows data gathering by both care providers and patients. The second phase of the project would be to combine clinical assessment with pharmacological insight. It may be especially relevant for patient monitoring after discharge, given that mHealth EMA is a promising method for reporting the clinical effects of pharmacological management. It will provide momentary assessment of the effects of a drug, including subjective perception of patients’ quality of life and health outcomes [7]. We will also be able to assess these features in the near future. These systems are able to explain how clinical practice is sometimes ahead of available evidence and may help develop better practices and security. At this point, we were able to provide information about drug management under real conditions and highlight points of conflict between ideal and real practice using the Web application. Our project may support the integration of mHealth techniques in prescription management systems and the development of future CDSSs.

## Appendix

Multimedia Appendix 1 Proportion of antipsychotic polypharmacy compared with monotherapy.

Multimedia Appendix 2 Antipsychotic defined daily dose (DDD) when use in monotherapy versus polytherapy.

## Abbreviations

APP: antipsychotic polypharmacy
ATC: Anatomical Therapeutic Chemical
CDSS: clinical decision support system
DDD: defined daily dose
EHR: electronic health record
EMA: ecological momentary assessment
mHealth: mobile health
PDD: prescribed daily dose

## References

[1] DesRoches CM, Campbell EG, Rao SR, Donelan K, Ferris TG, Jha A, Kaushal R, Levy DE, Rosenbaum S, Shields AE, Blumenthal D (2008). Electronic health records in ambulatory care--a national survey of physicians. N Engl J Med.

[2] Wang SJ, Middleton B, Prosser LA, Bardon CG, Spurr CD, Carchidi PJ, Kittler AF, Goldszer RC, Fairchild DG, Sussman AJ, Kuperman GJ, Bates DW (2003). A cost-benefit analysis of electronic medical records in primary care. Am J Med.

[3] Schiff GD, Bates DW (2010). Can electronic clinical documentation help prevent diagnostic errors?. N Engl J Med.

[4] Koppel R, Kreda DA (2010). Healthcare IT usability and suitability for clinical needs: challenges of design, workflow, and contractual relations. Stud Health Technol Inform.

[5] Celi LA, Marshall JD, Lai Y, Stone DJ (2015). Disrupting electronic health records systems: the next generation. JMIR Med Inform.

[6] East ML, Havard BC (2015). Mental health mobile apps: from infusion to diffusion in the mental health social system. JMIR Ment Health.

[7] Nicholas J, Larsen ME, Proudfoot J, Christensen H (2015). Mobile apps for bipolar disorder: a systematic review of features and content quality. J Med Internet Res.

[8] Nhavoto JA, Grönlund A (2014). Mobile technologies and geographic information systems to improve health care systems: a literature review. JMIR Mhealth Uhealth.

[9] Holzinger A, Dehmer M, Jurisica I (2014). Knowledge discovery and interactive data mining in bioinformatics--state-of-the-art, future challenges and research directions. BMC Bioinformatics.

[10] Krysiak-Baltyn K, Nordahl PT, Audouze K, Jørgensen N, Angquist L, Brunak S (2014). Compass: a hybrid method for clinical and biobank data mining. J Biomed Inform.

[11] Celi LA, Zimolzak AJ, Stone DJ (2014). Dynamic clinical data mining: search engine-based decision support. JMIR Med Inform.

[12] Wright BM, Eiland EH, Lorenz R (2013). Augmentation with atypical antipsychotics for depression: a review of evidence-based support from the medical literature. Pharmacotherapy.

[13] Zhornitsky S, Rizkallah E, Pampoulova T, Chiasson J, Stip E, Rompré P, Potvin S (2010). Antipsychotic agents for the treatment of substance use disorders in patients with and without comorbid psychosis. J Clin Psychopharmacol.

[14] Haw C, Stubbs J (2007). Off-label use of antipsychotics: are we mad?. Expert Opin Drug Saf.

[15] Montejo AL, Majadas S, Mayoral F, Sanjuán J, Ros S, Olivares JM, Gonzalez-Torres MA, Bousoño M (2006). [Analysis of prescription patterns of antipsychotic agents in psychiatry]. Actas Esp Psiquiatr.

[16] Connolly A, Taylor D (2014). Factors associated with non evidence-based prescribing of antipsychotics. Ther Adv Psychopharmacol.

[17] Linden M, Scheel T, Xaver EF (2004). Dosage finding and outcome in the treatment of schizophrenic inpatients with amisulpride. Results of a drug utilization observation study. Hum Psychopharmacol.

[18] Correll CU, Gallego JA (2012). Antipsychotic polypharmacy: a comprehensive evaluation of relevant correlates of a long-standing clinical practice. Psychiatr Clin North Am.

[19] López de Torre A, Lertxundi U, Hernández R, Medrano J (2012). Antipsychotic polypharmacy: a needle in a haystack?. Gen Hosp Psychiatry.

[20] Batalla A, Undurraga J, Grande I, Pons A (2010). P03-321 Antipsychotic polipharmacy in schizophrenic inpatients. Eur Psychiatry.

[21] Stahl SM (2013). Emerging guidelines for the use of antipsychotic polypharmacy. Rev Psiquiatr Salud Ment.

[22] Stahl SM, Grady MM (2004). A critical review of atypical antipsychotic utilization: comparing monotherapy with polypharmacy and augmentation. Curr Med Chem.

[23] Taylor D, Paton C, Kapur S (2012). The Maudsley Prescribing Guidelines in Psychiatry.

[24] Gaviria AM, Franco JG, Aguado V, Rico G, Labad J, de Pablo J, Vilella E (2015). A non-interventional naturalistic study of the prescription patterns of antipsychotics in patients with schizophrenia from the Spanish province of Tarragona. PLoS One.

[25] Hersh WR, Weiner MG, Embi PJ, Logan JR, Payne PR, Bernstam EV, Lehmann HP, Hripcsak G, Hartzog TH, Cimino JJ, Saltz JH (2013). Caveats for the use of operational electronic health record data in comparative effectiveness research. Med Care.

[26] Procyshyn RM, Honer WG, Wu TK, Ko RW, McIsaac SA, Young AH, Johnson JL, Barr AM (2010). Persistent antipsychotic polypharmacy and excessive dosing in the community psychiatric treatment setting: a review of medication profiles in 435 Canadian outpatients. J Clin Psychiatry.

[27] Lochmann van Bennekom MW, Gijsman HJ, Zitman FG (2013). Antipsychotic polypharmacy in psychotic disorders: a critical review of neurobiology, efficacy, tolerability and cost effectiveness. J Psychopharmacol.

[28] Fleischhacker WW, Uchida H (2014). Critical review of antipsychotic polypharmacy in the treatment of schizophrenia. Int J Neuropsychopharmacol.

[29] Ketter TA, Miller S, Dell'Osso B, Calabrese JR, Frye MA, Citrome L (2014). Balancing benefits and harms of treatments for acute bipolar depression. J Affect Disord.

[30] Kapur S, Zipursky R, Jones C, Remington G, Houle S (2000). Relationship between dopamine D(2) occupancy, clinical response, and side effects: a double-blind PET study of first-episode schizophrenia. Am J Psychiatry.

[31] van Doormaal JE, van den Bemt PM, Zaal RJ, Egberts AC, Lenderink BW, Kosterink JG, Haaijer-Ruskamp FM, Mol PG (2009). The influence that electronic prescribing has on medication errors and preventable adverse drug events: an interrupted time-series study. J Am Med Inform Assoc.

