# Supplementary material for: Ecological Assessment of Clinicians’ Antipsychotic Prescription Habits in Psychiatric Inpatients: A Novel Web- and Mobile Phone–Based Prototype for a Dynamic Clinical Decision Support System
Source: J Med Internet Res. 2017 Jan 26;19(1):e25. doi: 10.2196/jmir.5954 (PMC5301080; doi:10.2196/jmir.5954)
Supplement: Multimedia Appendix 1 [file jmir_v19i1e25_app1.pdf]

Supplementary Material 1: Proportion of antipsychotic polypharmacy compared with monotherapy

**Drug**	**ATC code**	**Total prescriptions**	**Monotherapy**<br><br>**Percentage (n)**	**Politherapy**<br><br>**Percentage (n)**
Amisulpride	N05AL05	17	29.4% (5)	70.6% (12)
Aripiprazole	N05AX12	37	67.6% (25)	32.4% (12)
Asenapine	N05AH05	25	72 % (18)	28%5 (7)
Clotiapine	N05AH06	11	18.2% (2)	81.8% (9)
Clozapine	N05AH02	11	18.2% (2)	81.8% (9)
Fluphenazine	N05AB02	3	0	100% (3)
Haloperidol	N05AD01	7	28.6% (2)	71.4% (5)
Levomepromazine	N05AA01	3	33.3% (1)	66.7% (2)
Olanzapine	N05AH03	36	52.8% (19)	47.2% (17)
Paliperidone	N05AX13	22	59.1% (13)	40.9% (9)
Long-acting paliperidone	N05AX13	63	42.9% (27)	57.1% (36)
Quetiapine	N05AH04	38	50% (19)	50% (19)
Risperidone	N05AX08	66	57.6% (38)	42.4% (28)
Long-acting risperidone	N05AX08	1	0	100% (1)
Tiapride	N05AL03	10	70% (7)	30% (3)
Ziprasidone	N05AE04	1	0	100% (1)
Zuclopenthixol acufase	N05AF05	2	0	100% (2)
Zuclopenthixol depot	N05AF05	12	16.7% (2)	83.3% (10)
Total		365	180	185
