# Supplementary material for: Ecological Assessment of Clinicians’ Antipsychotic Prescription Habits in Psychiatric Inpatients: A Novel Web- and Mobile Phone–Based Prototype for a Dynamic Clinical Decision Support System
Source: J Med Internet Res. 2017 Jan 26;19(1):e25. doi: 10.2196/jmir.5954 (PMC5301080; doi:10.2196/jmir.5954)
Supplement: Multimedia Appendix 2 [file jmir_v19i1e25_app2.pdf]

Supplementary Material 2: Antipsychotic DDD when use in monotherapy versus polytherapy

		One antipsychotic		Two antipsychotics		Three antipsychotics	
**Drug**	**ATC code**	**n**	**Mean PDD (mg)**	**n**	**Mean PDD (mg)**	**n**	**Mean PDD (mg)**
Amisulpride	N05AL05	5	840.00	11	781.82	1	1000.00
Aripiprazole	N05AX12	25	21.00	10	21.00	2	12.50
Asenapine	N05AH05	18	13.89	5	18.00	2	10.00
Clotiapine	N05AH06	2	25.00	5	44.00	4	30.00
Clozapine	N05AH02	2	250.00	8	381.25	1	250.00
Fluphenazine	N05AB02	0	-	3	33.33	-	-
Haloperidol	N05AD01	2	10.20	4	7.00	1	5.00
Levomepromazine	N05AA01	1	50.00	2	62.50	0	-
Olanzapine	N05AH03	19	15.79	14	14.82	3	20.00
Paliperidone	N05AX13	13	12.92	7	8.57	2	13.50
Long-acting paliperidone	N05AX13	27	121.30	28	145.54	8	137.50
Quetiapine	N05AH04	19	298.68	15	133.33	4	250.00
Risperidone	N05AX08	38	6.09	22	8.91	6	12.50
Long-acting risperidone	N05AX08	0	-	-	-	1	100.00
Tiapride	N05AL03	7	300.00	3	266.67	0	-
Ziprasidone	N05AE04	0	-	-	-	1	120.00
Zuclopenthixol acufase	N05AF05	0	-	2	50.00	-	-
Zuclopenthixol depot	N05AF05	2	200.00	7	200.00	3	266.67
Total		180	-	146	-	39	
